# Effect of Treatment Regimen on Long-Term Mortality of Geriatric Patients Diagnosed With Stable Coronary Artery Disease

**DOI:** 10.7759/cureus.13618

**Published:** 2021-02-28

**Authors:** Uğur Küçük, Ali Duygu, Bahadır Kırılmaz

**Affiliations:** 1 Cardiology, Canakkale Onsekiz Mart University Faculty of Medicine, Çanakkale, TUR; 2 Cardiology, Canakkale Onsekiz Mart University Faculty of Medicıne, Çanakkale, TUR

**Keywords:** geriatric, mortality, coronary artery bypass graft surgery

## Abstract

Background

Increased life expectancy across the world has resulted in an increase in the proportion of the elderly population who are lost to heart diseases. Advanced age and comorbidities are believed to change the response to treatments. In this study, we aimed to investigate the effects of surgical and medical treatments on the mortality of stable coronary diseases.

Methods

A total of 150 geriatric patients who underwent coronary angiography (CAG) were followed up in our cardiology clinic. Patients who decided to undergo coronary artery bypass graft (CABG) surgery after CAG and were willing to undergo the operation were assigned to group 1, whereas those who were unwilling to undergo the operation were not eligible for percutaneous coronary intervention and were followed up medically, and were assigned to group 2. Keeping the primary goal as mortality rates, both the groups were compared using medical records for three years after the treatment.

Results

After three years, the overall mortality rate included six patients (16%) in the CABG group versus 63 patients (55%) in the medical therapy group (p < 0.001). The CABG therapy was found to be significantly and independently associated with first- and third-year mortality (risk ratio: 0.064, 95% confidence interval: 0.009-0.467, p = 0.007; risk ratio: 0.305, 95% confidence interval: 0.151-0.615, p < 0.001, respectively). Kaplan-Meier analysis for first- and third-year all-cause mortality rates led to significant results and curves between the groups.

Conclusion

Our study revealed that compared to CABG surgery in the treatment of coronary artery disease in geriatric patients, medical treatment is associated with poor outcomes in terms of mortality in long-term follow-up.

## Introduction

Coronary artery disease (CAD) is an important cause of morbidity and mortality, especially in the elderly, as also in adults [1]. Advances in healthcare have resulted in an increase in life expectancy and an increase in the number of hospital admissions for elderly individuals in need of medical care. Since risk factors such as hypertension (HT), diabetes mellitus (DM), and obesity are more common in advanced ages, an increase in the prevalence of CAD has been observed worldwide [2].

Comorbid conditions, deterioration in cognitive functions, and atypical observation or late onset of symptoms in patients may cause some delays in treatment. While the guideline recommendations are mostly aimed at individuals under the age of 75 years, the underrepresentation of individuals of age >75 years in studies has led to a lack of opinion on the diagnosis and treatment of diseases, as frequently observed in older ages [3].

In geriatric patients, personalized treatment regimens are prominent due to factors such as the patient’s biological age, comorbid conditions, and intolerance to the applied treatment [4]. Risk scores such as Syntax, Gensini, and Grace, which are used in treatment management after coronary angiography (CAG), are used in patients diagnosed with CAD [5]. Several studies have suggested that the choice of an effective treatment method can reduce mortality and morbidity [6]. However, the ages of the patients have not been taken into consideration in the previous studies [7].

In our study, we aimed to investigate the importance of the treatment regimen to be selected and its effect on the mortality rate, irrespective of the age at which the treatment decisions of geriatric patients with stable CAD were taken.

## Materials and methods

The study included 150 patients of age >75 years who had three-vessel coronary artery stenosis in CAD and were hospitalized with the diagnosis of CAD between November 2009 and February 2018 at the cardiology clinic of the university hospital. Medical records include documentation of patient's history, clinical findings, diagnostic test results, operation notes, and death. In this retrospective study, the patients were divided into two groups. Group 1 included 38 patients who underwent coronary artery bypass operation. Group 2 included 112 patients who were offered coronary artery bypass surgery but refused to undergo the same. They were at high risk for percutaneous coronary intervention but did not accept interventional treatment and received medical treatment. Laboratory and CAG data were evaluated for both groups. The mortality rates in the two groups were compared using medical records during the one- and three-year follow-up period after angiography. The hemogram, lipid panel, and kidney function tests of all the patients were obtained from the patient’s medical file archive.

Exclusion Criteria

Patients of age <75 years who had chronic renal failure, acute coronary syndrome, active infection, malignant disease, cerebrovascular disease, and coronary artery bypass surgery, those who were scheduled for serious valve surgery, and those who had contraindications but did not accept CAG were not included in the study.

Before initiating the study, approval was obtained from the local ethics committee of the hospital, in line with the recommendations of the Declaration of Helsinki (Decision no: 2020-12).

Coronary Angiography and Calculation of Syntax Score

The standardized Judkins technique was used while CAG was performed through the femoral or radial arteries. At least two different image recordings were evaluated while deciding on the severity of the coronary artery. Lesions of ≥50% in at least one coronary artery were accepted as significant stenosis. The Syntax score was calculated using the most recent system (www.syntaxscore.com) for vessels with stenosis and diameter ≥1.5 mm above the p-value [8]. Two experienced cardiologists evaluated the angiography images of the patients and calculated their Syntax scores.

Statistical Analyses

SPSS 21.0 (SPSS Inc., Chicago, IL, USA) software was used for statistical analyses. Whether the variables fit the normal distribution was evaluated using the Kolmogorov-Smirnov test. While continuous variables were expressed as mean ± standard deviation, categorical variables were expressed as percent and numbers. A chi-square test was used to compare the probability ratios of categorical variables. Mann-Whitney’s U-test was used for data that did not show normal distribution. The Kaplan-Meier curves were used to indicate death in two patient groups, as defined in the medical and coronary artery bypass graft (CABG) treatment groups. Logistic regression analysis was performed to determine the effect of variables. In addition, 95% confidence intervals were calculated with standardized beta coefficients, and a p-value <0.05 was considered to be statistically significant.

## Results

**Figure 1:**
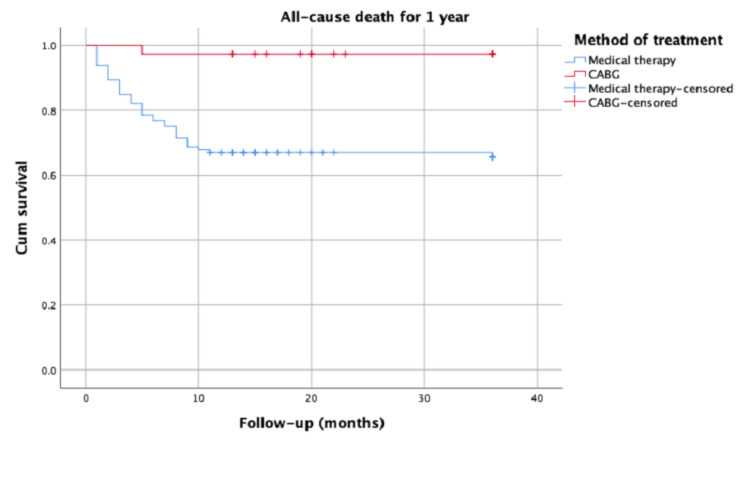
Kaplan-Meier analysis for all-cause mortality at first year CABG: coronary artery bypass grafting

**Figure 2:**
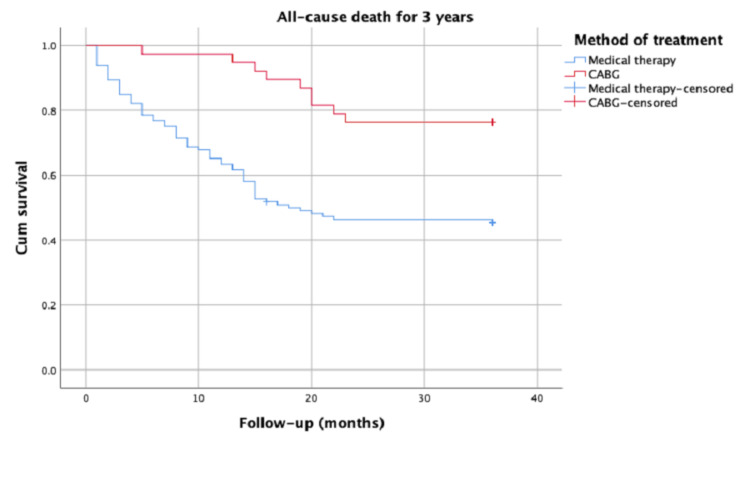
Kaplan-Meier analysis for all-cause mortality at third year CABG: coronary artery bypass grafting

The basic clinical characteristics, laboratory values, and pre-admission medications of the patient groups we enrolled in the study are listed (Table 1). The mean age of the patients included in the study was 79 ± 3 years. Of the 150 patients, 91 were men, and 59 were women. Only 38 of the geriatric patients were operated on, and 112 patients were followed up medically. The mean Syntax risk score was 24 ± 6, and there was no statistically significant difference between the groups (p = 0.290; Table 1). The echocardiographic data of the patients at the time of admission were not significantly different between the groups (p = 0.081; Table 1). Operation data are reported in Table 1. Cross-clamping time was 74.6 ± 6.92 minutes and cardiopulmonary bypass time was 112.43 ± 16.91 minutes. The median length of stay in intensive care unit was 7 days. The median intubation time was 26 hours. Postoperative resternotomy occurred in three patients due to tamponade. Blood transfusion was done in four patients. There were no in-hospital deaths in the on-pump CABG group. The mortality rate in the medical treatment group was statistically higher than in the CABG group (p < 0.001; Table 1). In univariate and multivariate logistic regression analyses, an independent relationship was established between the hemoglobin levels of the patients and the preferred treatment method (on-pump CABG) and first- and third-year mortality (Tables 2 and 3). The Kaplan-Meier analysis was performed for all-cause mortality at first and third years of patients with advanced-age stable CAD in medical and CABG treatments, with significant results (p < 0.001; Figures 1, 2).

**Table 1:** Baseline clinical characteristics, laboratory values, and preadmission medications of the patients CABG: coronary artery bypass grafting; WBC: white blood cell; DM: diabetes mellitus; HT: hypertension; LVEF: left ventricle ejection fraction; ACEI: angiotensinogen-converting enzyme inhibitor; ARB: angiotensin receptor blocker; ASA: acetylsalicylic acid; LDL-C: low-density lipoprotein cholesterol; Syntax: the synergy between percutaneous coronary intervention with taxus and cardiac surgery; LDL: low-density lipoprotein; CPB: cardiopulmonary bypass; IT: intubation time; ICU: intensive care unit

Variables	CABG (n = 38)	Medical therapy (n = 112)	p
Age (years)	78.8 ± 3.3	79.1 ± 3.1	0.623
Female n (%)	15 (10)	44 (29.3)	0.984
HT n (%)	24 (16)	60 (40)	0.304
DM n (%)	10 (6.7)	35 (23.3)	0.566
Systolic blood pressure (mmHg)	133.68 ± 9.13	133.15 ± 9.40	0.794
Diastolic blood pressure (mmHg)	77.32 ± 6.48	77.17 ± 7.58	0.925
Current smoker n (%)	13 (8.7)	58 (38.7)	0.061
Serum glucose (mg/dL)	127.42 ± 32.15	126.31 ± 32.40	0.855
Creatinine (mg/dL)	0.97 ± 0.27	1.11 ± 0.40	0.133
TSH (mU/L)	0.57 ± 0.43	1.76 ± 0.88	0.078
LDL-C (mg/dL)	120.29 ± 33.79	128.09 ± 39.09	0.148
Hemoglobin (g/dL)	12.18 ± 1.81	12.20 ± 1.84	0.795
WBC count (x10^3^/µL)	7.76 ± 2.10	8.19 ± 2.37	0.401
Neutrophil count (×10^3^/L)	5.14 ± 2.51	5.22 ± 2.31	0.803
Lymphocyte count (×10^3^/L)	1.74 ± 0.53	1.71 ± 0.59	0.927
Thrombocyte count (x10^3^/µL)	225.07 ± 68.84	223.99 ± 73.04	0.936
LVEF (%)	50.92 ± 8.5	48 ± 8.9	0.081
Syntax score	23.24 ± 6.10	24.48 ± 6.29	0.290
Medication n (%)			
ASA	28 (18.7)	85 (56.7)	0.785
ACEI/ARB	24 (16)	60 (40)	0.304
Beta blocker	5 (12.5)	14 (35)	0.873
Statin	6 (4)	25 (16.7)	0.390
Mean of death (year)	2.6 ± 0.7	1.7 ± 1.1	<0.001
Time of CPB (minutes)	112.43 ± 16.91		
Duration of cross-clamping (minutes)	74.6 ± 6.92		
IT (hours)	26 (2-55)		
ICU stay (days)	7 (3-12)		
Mortality n (%)			
1 year	1 (2.6)	38 (33.9)	<0.001
1-3 years	5 (13.5)	25 (33.8)	0.023

**Table 2:** Univariate and multivariate cox regression analyses for predicting first-year mortality Multivariate Cox proportional-hazards model including the variables in univariate analysis and also variables found to be significantly different between groups I and II with forward stepwise method. CI: confidence interval; HR: hazard ratio; LDL-C, low-density lipoprotein cholesterol; ACEI, angiotensinogen-converting enzyme inhibitor; ARB, angiotensin receptor blocker; ASA, acetylsalicylic acid; CABG, coronary artery bypass grafting

	Univariate			Multivariate		
Variables	p	HR	95% CI	p	HR	95% CI
Age	0.520	1.032	0.938-1.135			
Syntax score	0.358	1.024	0.973-1.077			
Diabetes mellitus	0.301	1.481	0.703-3.120			
Hypertension	0.830	1.072	0.569-2.019			
Hemoglobin levels	0.035	0.828	0.696-0.987	0.027	0.824	0.694-0.979
Creatinine	0.746	0.835	0.281-0.484			
Serum glucose	0.772	0.999	0.989-1.009			
LDL-C	0.286	1.004	0.996-1.012			
Usage of beta blocker	0.815	0.927	0.492-1.746			
Usage of ACEI/ARB	0.830	0.933	0.495-1.757			
Usage of ASA	0.960	0.982	0.478-2.014			
Usage of statin	0.993	0.996	0.458-2.168			
Treatment method (CABG)	0.007	0.065	0.009-0.475	0.007	0.064	0.009-0.467

**Table 3:** Univariate and multivariate cox regression analyses for predicting third-year mortality Multivariate Cox proportional-hazards model including the variables in univariate analysis and also variables found to be significantly different between groups I and II with forward stepwise method. CI: confidence interval; HR: hazard ratio; LDL-C: low-density lipoprotein cholesterol; ACEI: angiotensinogen-converting enzyme inhibitor; ARB: angiotensin receptor blocker; ASA: acetylsalicylic acid; CABG: coronary artery bypass grafting

	Univariate			Multivariate		
Variables	p	HR	95% CI	p	HR	95% CI
Age	0.311	1.038	0.966-1.116			
Syntax score	0.541	0.988	0.951-1.027			
Diabetes mellitus	0.472	1.214	0.717-2.055			
Hypertension	0.625	1.124	0.703-1.798			
Creatinine	0.498	0.738	0.307-1.775			
Serum glucose	0.880	0.999	0.992-1.007			
Hemoglobin levels	0.043	0.875	0.770-0.996	0.028	0.866	0.762-0.984
LDL-C	0.183	1.004	0.998-1.010			
Usage of beta blocker	0.648	0.895	0.558-1.438			
Usage of ACEI/ARB	0.830	0.933	0.495-1.757			
Usage of ASA	0.811	0.938	0.554-1.588			
Usage of statin	0.603	0.852	0.467-1.557			
Method of treatment (CABG)	0.001	0.314	0.156-0.633	0.001	0.305	0.151-0.615

## Discussion

Cardiovascular diseases top the list among the causes of death across the world. The main form of cause is atherosclerosis, and there may be various endpoints such as CAD, cerebrovascular diseases, or peripheral or carotid artery diseases [9].

As a result of the three-year follow-up in our study, we demonstrated that the prognosis of patients who underwent surgery was better than that of the medical group, where long-term follow-up of advanced-age patients diagnosed with stable CAD was undertaken.

As per the current guideline recommendation, the main purpose of treatment in patients with stable CAD is to prolong their life span, and the preference for surgery is low due to the presence of multivessel diseases in angiography performed in geriatric patients and the accompanying comorbid conditions, which is why medical or percutaneous coronary interventional procedures are more preferred [10]. In the Courage study, no differences were noted in terms of cardiac events between the groups that underwent medical treatment and percutaneous coronary intervention in patients diagnosed with chronic stable CAD of age >65 years [11].

The effect of invasive treatment on survival was statistically similar when compared to the medical treatment performed by Matthias et al. in geriatric patients of an average age of 80 years [12]. In another study, Azin et al. conducted long-term follow-ups in which chest pain and functional capacity were positively affected in the operated group of advanced-age patients who underwent CABG operation and who received medical treatments. The authors demonstrated that mortality rates after one month were similar between the groups [13]. Unlike in all these past studies, in the Kaplan-Meier statistical analyses performed for all-cause mortality in our study, geriatric patients showed better survival rates for both short-term one-year and long-term three-year survival analyses in the group that had the CABG operation.

Left ventricular ejection fraction (LVEF) is recognized as a strong predictor of survival in patients with cardiovascular diseases [14]. While the differences obtained in the studies performed were interpreted as being related to accompanying clinical manifestations (such as HT and DM) and the differences in LVEF, no differences were noted between the groups for the variables specified in our study [15,16].

Syntax score, which is frequently used for treatment preferences after CAG, provides important information, especially in multivessel diseases, main coronary lesions, and complex cases [17]. Percutaneous coronary intervention or surgery as a treatment option, when performed according to the Syntax score in advanced age, provides a significant reduction in morbidity and mortality. In the study conducted by Madeleine et al., the Syntax score predicted the risk of death in patients of age >75 years in stable and unstable patients, limited to 1 year [18]. In the three-year follow-up in our study, the Syntax score was not useful in predicting the risk of death among the advanced-age treatment groups. Contrary to that in past studies, the Syntax score was calculated in our study by only looking at the coronary anatomy in elderly patients, which is not sufficient, necessitating making a personalized decision by evaluating other accompanying clinical manifestations.

The most significant limitation of our study is that it is a single-center and retrospective study. Another limitation is the small number of individuals included in our study, and the most important reason for this is that the desired data could not be accessed. However, the prospective follow-up of a large number of patients of age >75 years who had undergone CABG or medical treatment is difficult and a time-consuming task.

## Conclusions

In conclusion, considering the technological methods developed in the field of coronary artery bypass surgery and the experience of the surgical team, it should be kept in mind that surgical treatment positively affects long-term outcomes when compared to medical treatments provided to geriatric patients.
